# Integrative nuclear and total proteomics of compound leaves in semi-leafless and leafed pea cultivars reveal regulatory cues in leaf development

**DOI:** 10.1186/s12870-026-08381-5

**Published:** 2026-03-04

**Authors:** Rajiv K. Tripathi, Arun Kommadath, Ravinder K. Goyal

**Affiliations:** Agriculture and Agri-Food Canada, Lacombe Research and Development Centre, Lacombe, AB T4L 1W1 Canada

**Keywords:** Biological processes, Leaf development, Nuclear proteomics, Pea (*Pisum sativum* L.), Protein–protein-interactions, Redox homeostasis, Tendril development, Transcription factors

## Abstract

**Background:**

Leaf architecture is a critical determinant of crop productivity, influencing light interception, canopy organization, and lodging tolerance. In pea (*Pisum sativum* L.), the semi-leafless phenotypes with well-developed tendrils improved the standability of the crop which resulted in better photosynthetic ability of the canopy and yield, reduced losses to diseases, and improvement in seed quality. These traits associated with semi-leafless cultivars have transformed modern breeding programs. The underlying molecular networks governing compound leaf development, however, remain poorly understood. Here, we present an integrative proteomic atlas of pea leaf tissues, generated by profiling both nuclear and total proteomes of semi-leafless (Cooper) and leafed (Trapper) cultivars.

**Results:**

Quantitative analyses identified more than 8,500 nuclear and 7,700 total proteins across tendrils, stipules, leaflets, rachis, and petioles. Comparative profiling revealed stronger conservation in nuclear proteomes (~ 37% differentially abundant proteins) than in total proteomes (~ 78%), with young tissues contributing disproportionately to proteomic variation. Tissue-and cultivar-specific differences were pronounced. The genetic variation at the *afila* locus strongly influenced tendril proteomes, with Cooper displaying widespread proteome reprogramming consistent with its highly branched phenotype, whereas Trapper’s residual tendril development was heavily shaped by nuclear regulation. Proteins associated with transcription factor families, including WDR, C2H2, bZIP, PHD, TF-B3, redox-homeostasis regulators, phytohormone signaling, chromatin modulators, and proteins regulating cell structure emerged as important contributors to different leaf developmental pathways. Additionally, clusters of uncharacterized proteins with unique abundance patterns across tissues and cultivars point to potential roles in organ identity and regulatory complexity yet to be uncovered.

**Conclusions:**

Together, these results provide unparalleled resolution of the proteomic landscape underlying pea leaf development. The resource offers novel insights into the regulatory complexity of compound leaf formation and establishes a foundation for systems-level approaches for molecular understanding.

**Supplementary Information:**

The online version contains supplementary material available at 10.1186/s12870-026-08381-5.

## Introduction

Pea (*Pisum sativum* L.) is among the most important legume crops cultivated worldwide for both food and feed purposes. Owing to its high protein content, pea is increasingly valued as an alternative source of plant-based protein. Its ability to fix atmospheric nitrogen not only supports self-sufficiency in nitrogen but also reduces fertilizer requirements for subsequent crops, thereby lowering greenhouse gas emissions [1, 2]. Enhanced crop productivity, to which leaf function is a key contributor, remains a primary goal in plant breeding. As the main organ of light interception, the leaf plays central roles in photosynthesis, respiration, and transpiration, with its shape, size, and canopy architecture directly influencing productivity [3]. Beyond these core functions, leaves are modified for specialized roles, such as water storage in succulents, spines for defense, or tendrils for climbing and support. Leaves also integrate developmental signals that mediate the transition from vegetative to reproductive growth [4]. In pea, the leaf is pinnately compound, comprising basal foliaceous stipules, proximal leaflets, and distal tendrils [5].

Several pea mutants have been instrumental in elucidating the genetic regulation of leaf morphology. *Stamina pistilloida* (*stp*) and *unifoliata* (*uni*) mutants alter leaf phyllotaxy and pinnation [6–8], while *crispa* (*cri*) affects lamina length, shape, and polarity [9]. The *tendril-less* (*tl*) mutation converts tendrils into leaflets, suggesting that tendril is a form of leaflet [8]. A drastic reduction in stipule size was observed in *stipule reduced *(*st*) mutants [10]. These mutants not only revealed key regulators of leaf morphogenesis but also influenced pea breeding. The *afila* (*af*) mutation, which replaces the leaflets with tendrils, significantly impacted pea cultivation. Excessive lodging reduces the photosynthetic capacity of the plant canopy resulting in poor yield, higher disease incidence and low seed quality due to plant’s contact with soil, and causes harvesting challenges, thus rendering the commercial cultivation of pea uneconomical. The development of semi-leafless cultivars with tendrils in the 1970 s overcame this limitation, and such varieties now account for 80–90% of pea cultivation in Europe and Canada [11], highlighting the significance of leaf architecture in varietal adoption.

Studies have identified critical genes underlying these morphological variants. Recently, it has been shown that the absence of *PALMATE-LIKE PENTAFOLIATA* (*PALM*) genes, *PsPALM1a* and *PsPALM1b*, in semi-leafless pea varieties is associated with the *af* phenotype [12, 13]. These genes are tandemly arranged on chromosome 2 of pea and encode a Q-type Cys(2)-His(2) zinc finger transcription factor. The *Tl* encodes a Class I homeodomain leucine zipper (HDZIP) transcription factor that suppresses the development of lamina [8], while *uni*, an ortholog of the LEAFY transcription factor, directs a simple leaf structure and converts flowers into vegetative structures [6]. The *st* gene encodes a C2H2 zinc finger transcription factor regulating stipule cell division and expansion, under the control of *Cochleata*, an ortholog of *Arabidopsis BLADE-ON-PETIOLE* [14, 15]. In addition, redox regulation plays a critical role in leaf development, as cysteine-mediated modifications in transcription factors modulate developmental programs [16, 17]. For instance, the DNA-binding activity of TCP and HD-ZIPIII transcription factors is redox-sensitive [16, 18, 19], whereas Kua1, a MYB-like transcription factor, regulates peroxidases to control leaf cell size [20].

Transcriptome analytical studies provided valuable insight into different aspects of leaf development [21–23]. Often, transcript levels are used as proxies for the protein abundance. They may not accurately represent the status of the proteins in cells or organs. While proteomics has been widely applied in other species, pea leaf development remains poorly characterized. Previous efforts focused primarily on seed proteomes, including identification of 156 proteins in mature seeds [24] and 2,195 proteins in comparative analyses of yellow versus green seeds [25]. Dynamic proteome changes in developing seeds under sulfur deficiency and water stress revealed > 3,000 proteins [26], while more recent mapping of round versus wrinkled pea seeds identified 3,659 proteins [27], representing the most comprehensive pea seed proteome to date. Proteome studies in pea lagged, largely due to the lack of a reference genome. Until recently, protein identification often relied on mapping to non-pea species [28]. With the release of high-quality genomes for pea cultivars Cameor and Zhongwan 6 [29, 30], coupled with advances in quantitative proteomics, a deeper understanding of pea leaf biology is now possible. Here, we present a quantitative proteomic atlas of leaf tissues from semi-leafless (Cooper) and leafed (Trapper) pea cultivars. By profiling both nuclear and total proteomes, we identified over 8,500 and 7,700 proteins, respectively. This dataset provides an unprecedented resource for dissecting the molecular underpinnings of leaf development and offers new insights into the systems-level regulation of compound leaf architecture in pea.

## Materials and methods

### Plant material and tissue collection

Pea (*Pisum sativum* L.) cultivars, Cooper (CO; semi-leafed) and Trapper (TR; leafed), were used in the study. The seeds of these cultivars were obtained from the seed store of AAFC-Lacombe Research and Development Center’s pea breeding program. Seeds were planted in 2.84 L pots containing Sungro-Sunshine mix (Agawam, MA, USA) supplemented with rhizobia and Harrell’s Pro-fertilizer. Plants were grown in a controlled environment chamber at the Lacombe Research and Development Center (Alberta, Canada) under a 16 h light/8 h dark cycle, with day/night temperatures of 22 °C/18 °C, relative humidity of 50–60%, and light intensity of 263 µmol m⁻^2^ s⁻^1^. Plants were watered as required. Compound leaf tissues were dissected into young tendril (YT), mature tendril (MT), young stipule (YS), mature stipule (MS), rachis (R), petiole (P) and Trapper leaflets (LF), which are absent in Cooper. YT samples were collected at the earliest possible developmental stage, yielding ~ 2 mg fresh weight per plant in Trapper and ~ 12 mg in Cooper. Although the seedlings were 22-days old, the tissues when analyzed were not at the advanced developmental stage, but rather nascent tendrils collected soon after their differentiation from the terminal apex branch. The mature tissues were collected from the 5-6th and 7-8th nodes of Cooper and Trapper, respectively. The difference in nodes was due to faster growth in Trapper than Cooper. The mature tissues were defined as fully expanded organs although tendril growth and development did not cease completely, especially in Cooper, at the sampling stage. All tissues were harvested in three biological replicates, flash frozen in liquid nitrogen, and stored at −80 °C until use.

### Total protein extraction and quantification

Total proteins were extracted using Pierce plant total protein extraction kit (Thermo Scientific) following the manufacturer’s protocol. Briefly, 100 mg of frozen tissue was homogenized in 100 µL of 1 × lysis buffer supplemented with Halt Protease Inhibitor Cocktail (Thermo Scientific). Samples were incubated at room temperature for 3 min and centrifuged at 13,000 rpm for 5 min. The supernatant containing total proteins was collected and stored at –80 °C until further use.

Protein concentration was determined using the Pierce BCA Protein Assay Kit (Thermo Scientific) following the microplate procedure. Ten µL of standard or sample was dispensed into wells of a 96-well plate, followed by addition of 200 µL of working reagent. After shaking for 30 s, plates were covered with aluminum foil and incubated at 37 °C for 30 min, cooled to room temperature, and absorbance was measured at 562 nm using a BioTek Synergy HTX microplate reader (Agilent). Protein concentrations were calculated using a bovine serum albumin (BSA) standard curve.

### Nuclei isolation

Nuclei were isolated from frozen leaf tissues using a modified protocol [31]*.* Three grams of each sample was ground to fine-powder in liquid nitrogen using mortar and pestle and resuspended in 10 volumes of 1 × nuclei isolation buffer (NIB); 10 mM MES-KOH (pH 5.4), 10 mM NaCl, 10 mM KCl, 2.5 mM EDTA, 250 mM sucrose, 0.1 mM spermine, 0.5 mM spermidine, 1 mM DTT, 0.1% protease inhibitor, and 1% polyvinylpyrrolidone. The homogenates were filtered through two layers of pre-wetted cheesecloth and one layer of pre-wetted Miracloth in 1 × NIB. Triton X-100 (10%) was added to the solution to a final concentration of 0.5% and agitated at 80–90 rpm at 4^0^C for 20 min. The homogenate was centrifuged at 1000 × g for 10 min followed by resuspension of the pellet in 10 ml of NIB. Crude nuclei were separated using Percoll/sucrose gradient prepared by pipetting 5 ml of 2.5 M sucrose into a 15 ml falcon tube with overlaying 5 ml of 60% Percoll. The nuclei preparation was loaded on the gradient and centrifuged at 1000 × g for 30 min at 4^0^C. The nuclear fraction at the interface of Percoll and sucrose was collected, resuspended in 5 volumes of 1 × NIB and 0.5% Triton X-100, agitated for 10 min followed by centrifugation at 1000 × g for 10 min. The pellet was again resuspended in 5 ml of 1 × NIB and loaded by dropwise on top of the 35% Percoll solution in a 15 ml falcon tube and centrifuged at 1000 × g for 10 min. The nuclei pellet was washed in 5 ml of 1 × NIB, centrifuged and resuspended in 500 µl of nuclei storage buffer (20% glycerol, 20 mM HEPES–KOH (pH 7.2), 5 mM MgCl_2_, 1 mM DTT).

Nuclei quality was verified by staining with 4’,6-diamidino-2-phenylindole (DAPI). The stain was prepared by dissolving 1 µg/ml of DAPI in 1 × phosphate buffer saline (pH 7.4). Ten µl of nuclei preparation was mixed with 10 µl of DAPI solution and visualised on a Nikon Eclipse TE300 inverted microscope using the DAPI filter (Fig. S1A-B).

### DNA extraction and polymerase chain reaction (PCR)

It has been shown that deletion of *PsPALM1a* and *PsPALM1b* genes is associated with *afila* phenotype [12]. Therefore, these genes were PCR amplified in pea cultivars, Cooper and Trapper. DNA was extracted from fresh stipules using DNeasy Plant pro kit (Qiagen; Cat. No. 69206) according to the manufacturer’s instructions. The primer sequences used for PCR to amplify *PsPALM1a/1b* fragments were selected from a previous study [12].

*PsPALM1a-*forward: TCTCTGTCTTTTGCAGCGTGTAGTG.

*PsPALM1a-*reverse: TGCTATCAATTTCATGTATAGCTGG.

*PsPALM1b-*forward: TCTCACTTTTGCAGTGTGTAGTGAAG.

*PsPALM1b*-reverse: CCTATCAATTTCATAAAAAAAGCTAGC.

PCR was performed using 2X GoTaq master mix (Promega) using the following conditions; 95^0^C (1 min) (95^0^C [30 s]/53^0^C [30 s]/72^0^C [30 s]), X 36 cycles, 72^0^C (5 min), on a X50 mastercycler (Eppendorf). PCR products were visualized on 1.5% agarose gel.

### LC–MS/MS sample preparation

Protein sample preparation for LC–MS/MS analysis was performed following the protocol described by [32] with minor modifications at the Proteomics Core Facility, University of British Columbia (Vancouver, Canada). Briefly, 20 µg of total protein from each sample was resolved on 10% SDS-PAGE (one dimension) until the dye front migrated approximately 1–2 cm into the gel. Entire gel lanes were excised, cut into 1–2 mm fragments, and destained using a buffer containing 50 mM NH₄HCO₃ and ethanol (6:4, v/v), followed by dehydration in 100% ethanol. Disulfide bonds were reduced with 10 mM 1,4-dithiothreitol (DTT) at 56 °C for 45 min and alkylated with 55 mM iodoacetamide in the dark at room temperature for 30 min. The gel pieces were subjected to successive cycles of dehydration and rehydration in 50 mM NH₄HCO₃ (pH 8.0), then digested overnight at 37 °C with sequencing-grade trypsin. Digestion was quenched with 1% trifluoroacetic acid (TFA), and peptides were extracted twice with 50% acetonitrile (ACN)/0.1% TFA and twice with 100% ACN. Extracted peptides were concentrated and purified using STAGE-tips containing C18 material. Columns were conditioned and equilibrated with 0.1% TFA, washed twice with 0.1% TFA, and peptides were eluted with 40% ACN/0.1% TFA. Eluates were dried and reconstituted in 0.5% ACN/0.1% formic acid. Peptide concentrations were determined using NanoDrop One (Thermo Scientific) with the A205 method (absorbance at 205 nm; baseline correction at 340 nm).

### Liquid Chromatography (LC) and Mass-Spectrometry (MS)

For LC–MS/MS, 100 ng of reconstituted peptide digest was injected onto an Easy-nLC 1200 system (Thermo Fisher Scientific). The system was equipped with a 25 cm Aurora Series Gen2 analytical column (Ion Opticks, Parkville, Australia) maintained at 40 °C with an integrated column oven (PRSO-V2, Sonation, Germany). Buffer A consisted of 0.1% formic acid and 2% ACN in water. Buffer B was consisted of 0.1% formic acid and 80% ACN in water. Columns were equilibrated with four column volumes of buffer A prior to sample loading. A standard 60 min gradient was run from 2% B to 20% B over 46 min. After that 32% B was run over 15 min, followed by 50% B from 61 to 44 min, and 95% B over 5 min. The run was held at 95% B for 8 min. Further, it was dropped to 3% B over 2 min, and held at 3% B for 6 min. The flow rate was maintained at 0.25 µL/min, and the autosampler thermostat was set to 7 °C.

The peptides were analyzed with an Orbitrap Exploris 480 mass spectrometer (Orbitrap ExplorisTM 480, Thermo Fisher Scientific). The Nanospray FlexTM ion source was operated at 1900 V spray voltage and ion transfer tube was heated to 290 °C. During analysis, the Orbitrap Exploris 480 was operated in a data-independent (DIA) mode. The MS and MS/MS spectra were collected in positive mode. Full MS scans were acquired at a resolution of 60,000 with a normalized automatic gain control (AGC) target of 300%, RF lens setting of 50%, maximum injection time of 25 ms, and a scan range of m/z 380–985. DIA fragment spectra were collected at a resolution of 15,000, normalized AGC target of 2000%, maximum injection time of 40 ms, scan range from m/z 145 Th – m/z 1450 Th. Isolation windows of m/z 10 Th were used with an overlap of m/z 1 Th. Normalized collision energy was set at 28%.

### Data-independent acquisition neural network (DIA-NN) analysis

Raw spectral data were processed with DIA-NN (version 1.8.1) [33]. The *Pisum sativum* cv. Zhongwan 6 (ZW6) protein sequences (www.uniprot.org/) were used as reference to DIA-NN. The FASTA digest for library free search/library generation, and deep learning spectra were selected as parameters for the analysis. Trypsin/P was selected as protease specificity and missed cleavage was selected to one. Peptide length range was 7–30 amino acids, precursor charge ranged 1–4, precursor m/z ranged 300–1200, and fragment ion m/z ranged 200–1800. The robust LC (high precision) approach was used for quantification strategy, RT-dependent mode for cross-run normalization, and smart profiling mode for library generation.

### Analysis of differential abundance of proteins

Protein quantification values were grouped by tissue-type and analyzed using the Mass Dynamics 2.0 (MD2.0) software platform [34, 35]. Proteins identified solely by site, potential contaminants, and reverse-sequence analogs were excluded prior to statistical processing. Intensities were log₂-transformed, and missing values were imputed using a missing-not-at-random (MNAR) strategy with a mean shift of –1.8 and a standard deviation of 0.3. Differential protein abundance was assessed in R (v4.2.0; www.r-project.org) using the Bioconductor (v3.16; www.bioconductor.org) package limma (v3.54.2) [36]. Linear models with robust empirical Bayes moderation were applied to compute moderated *t*-statistics, and *p*-values were adjusted for multiple testing using the Benjamini–Hochberg method. Only proteins with ≥ 50% valid measurements in a given comparison were considered, and those with adjusted *p*-values (*p*adj) below 0.05 were defined as differentially abundant proteins (DAPs). Intensity heatmaps were generated through seaborn clustermap (v0.11.2) [37] in MD2.0 platform. Protein log₂ intensities (original or imputed) were Z-score normalized, and hierarchical clustering was conducted using the Euclidean distance metric. The word cloud (www.wordclouds.com/) was generated using the numbers of orthologues of leaf development proteins extracted from ANOVA identified DAPs.

### Gene ontology enrichment, and protein-network analysis

To characterize functional shifts associated with pea compound leaf development, gene ontology (GO) enrichment analysis was performed on gene IDs corresponding to DAPs from each tissue comparison. Gene annotations for the pea cultivar ZW6 were retrieved from the “ZW6.annotation.gff3” file (downloaded July 5, 2024) from the pea genome database (www.peagdb.com/download/). GO term mappings provided by Dr. Tao Yang [30] were used as the primary annotation source. Because some terms were deprecated in the updated GO database release (2024–06–17; geneontology.org) used at the time of this analysis, alternative terms were identified and updated using the Bioconductor package GO.db (v3.19.1) [38], and their definitions were mapped wherever possible. The *gene* IDs corresponding to protein IDs in each tissue contrast studied were tested for enriched GO terms using the *enricher* function from the Bioconductor (version 3.19; www.bioconductor.org/) package *clusterProfiler* version 4.12.0 [39]. Parameters included a minimum of three annotated proteins per biological process, a background gene set comprising all annotated genes from the respective tissue contrast, and a Benjamini–Hochberg corrected significance threshold of *p*adj < 0.05. Both over-represented and under-represented biological processes were identified based on false discovery rate (FDR)-adjusted values.

Protein–protein interaction networks were constructed using STRING (v12.0; string-db.org). At present, a curated pea (*Pisum sativum*) protein–protein interaction network is not available in the STRING database as well as its genome is not well annotated. Therefore, the legume model species *Medicago truncatula*, which is phylogenetically closely related to pea and has a well-annotated interaction dataset, was used as a reference to infer putative protein–protein interactions. Amino acid sequences of up-regulated proteins from each tissue-type comparison were queried against *Medicago truncatula* as the reference organism in the STRING database. For network construction, proteins significantly up-regulated in CO-MT vs TR-LF (*p*adj = 0.0312), CO-YS vs CO-MS (*p*adj = 0.0485) in the total proteome, and TR-YS vs TR-MS in both total (*p*adj = 0.0281) and nuclear (*p*adj = 0.0217) proteomes were used. To accommodate the STRING input limit, proteins were ranked by adjusted *p*-value, and only the top 2000 proteins per comparison were included. The parameters used to develop the protein-network were set to develop a full string network with 0.7 high confidence score utilizing all the active interaction sources available from text mining, experiments, databases, co-expression, neighborhood, gene fusion, and co-occurrence. Disconnected proteins were excluded from the network display. Further, *k*-means clustering was applied to identify protein groups enriched with specific processes in each network. The tab separated values file from STRING was exported to Cytoscape version 3.10.3 [40]. Node scores were calculated using Cytoscape plugin *cytohubba* to identify hub proteins [41]. The Maximal Clique Centrality (MCC) method was used to identify the top 10 nodes. UpSet (intersect) plots were generated in R using the *UpSetR* function with a subset size of ten.

## Results

### A protein atlas of leafed and semi-leafless pea cultivar leaves

**Fig. 1:**
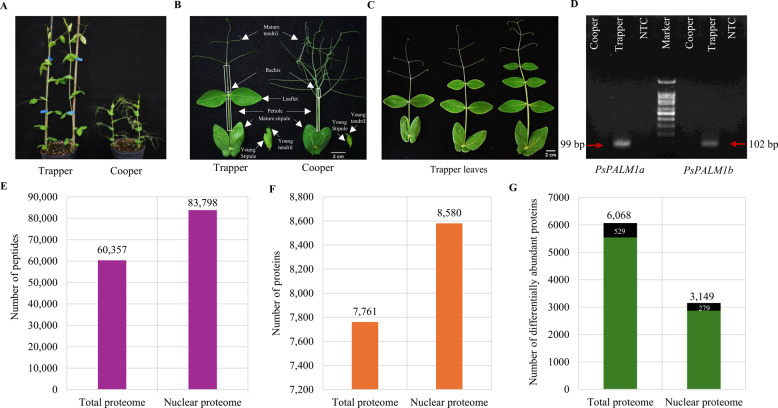
Phenotypic and proteomic features of Cooper and Trapper pea cultivars. **A** Twenty-two-day-old Cooper and Trapper plants showing distinct plant and leaf morphologies. **B** Representative compound leaves of Trapper and Cooper, highlighting tissue types and the young tissues sampled for proteome analyses. **C** Trapper leaves at different developmental stages showing one, two, or three pairs of leaflets; leaves with a single leaflet pair at the node 8 stage were used for proteomics. **D** PCR validation of *PsPALM1a* and *PsPALM1b* genes, as shown by red arrows, in Trapper and Cooper; marker represents a 100-base pair (bp) DNA ladder and NTC denotes a non-template control. Full-length original gel image is provided in Fig. S1C. **E**–**G** Summary of proteomic analyses showing the number of detected peptides **E**, proteins **F**, and differentially abundant proteins **G** in total and nuclear proteomes. In panel **G**, shaded bars and values indicate uncharacterized proteins

To obtain a comprehensive view of total and nuclear proteomes in compound leaves of leafed and semi-leafless pea cultivars, we conducted an extensive proteomic analysis. The study compared Trapper (leafed) and Cooper (semi-leafless) (Fig. 1A), which differ in leaf architecture and compound leaf morphology. Cooper, lacking a pair of leaflets, exhibited shorter rachis and highly branched tendrils compared with Trapper (Fig. 1B). Trapper leaves developed one to three pairs of leaflets during growth (Fig. 1C). Recent studies linked the semi-leafless trait to deletions of *PsPALM1a* and *PsPALM1b* genes associated with *af* locus [12, 13]. To confirm that Cooper shares this genetic background, PCR analysis on genomic DNA from stipules amplified *PsPALM1a/1b* fragments in Trapper but not in Cooper (Fig. 1D; Fig. S1C). For proteome analyses, compound leaves were dissected into six tissue types: YS, MS, P, R, YT, and MT, with Trapper including an additional leaflet (LF) absent in Cooper (Fig. 1B). Correlation among replicates of the same tissue ranged from 0.8 to 0.9 (Fig. S2A-B). Across three biological replicates per tissue, a total of 60,357 peptides representing 7,761 total proteins and 83,798 peptides corresponding to 8,580 nuclear proteins (Fig. 1E-F; Tables S1, S2) were identified. Each tissue yielded 6,000–7,000 total proteins, except TR-MS and TR-LF (Fig. S2C), while nuclear proteomes contained 6,200–7,400 proteins, excluding stipules (Fig. S2D). The proteins were identified from different sizes of peptides ranging from 7 (minimum), 12 (median) and 30 (maximum) amino acids in both the proteome studies. Unexpectedly, more nuclear than total proteins were identified. It might possibly be due to differential protein intensities, which showed a flatter concentration gradient in nuclear proteins, producing more even signal intensities. In contrast, the broader range of total protein intensities effectively raised the detection threshold of mass spectrometry, thereby reducing the number of protein identifications. Factors such as concentration range, turnover, proteome dynamics, and peptide physicochemical properties influence LC–MS detection limits [42]. While it was not possible to determine the precise representation of nuclear proteins within the total proteome, less abundant nuclear proteins were likely underrepresented.

**Fig. 2:**
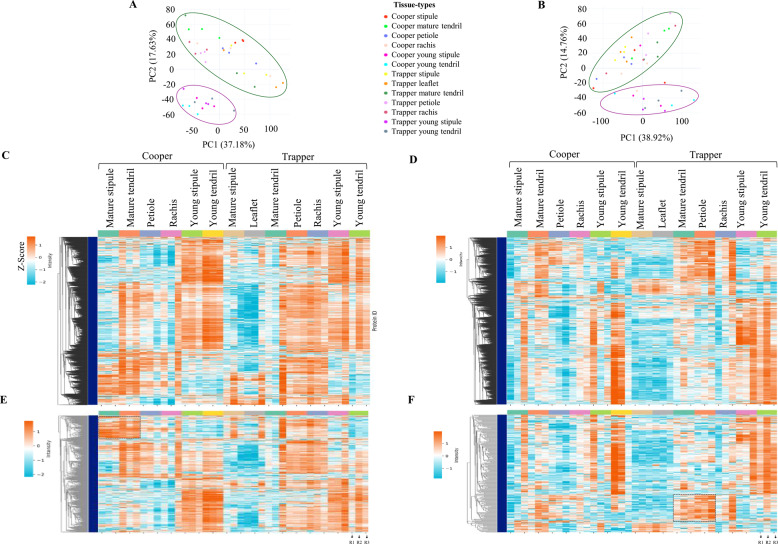
Proteins clustering by Principal Component Analysis (PCA) and protein intensity heatmaps. **A** PCA plot of total proteomes, and **B** nuclear proteomes, showing separation by leaf stage and tissue-type. Green and pink circles denote mature and young tissue, respectively. **C** Intensity heatmap of the proteome profile of 6,068 total, and **D** 3,149 nuclear proteins (Table S3) across 39 biological replicates of 13 pea leaf tissue-types. **E** Abundance patterns of 529 uncharacterized proteins in total, and **F** 279 uncharacterized nuclear proteins. The rectangles highlight cluster of proteins discussed in the results. Log2 intensities (original or imputed) of differentially abundant proteins (Benjamini–Hochberg corrected *p* < 0.05 from ANOVA) are displayed. Columns represent leaf tissue and rows as proteins. The pseudocolor scale (cadmium orange and blue showing higher and lower abundance, respectively) represents normalized Z scores of protein intensities. Dendrograms represent unsupervised agglomerative hierarchical clustering with a cluster distance of 15. R1, R2 and R3 indicate biological replicates

Principal Component Analysis (PCA) of both proteomes clearly separated young and mature tissues (Fig. 2A-B). ANOVA identified 6,068 total and 3,149 nuclear proteins as differentially abundant, highlighting lesser variation among nuclear proteins (Fig. 1G; Table S3). Of these, 529 total and 279 nuclear proteins remain uncharacterized. We performed domain analysis of uncharacterized proteins and identified putative domains in some proteins, many of these predictions were partial or incomplete and did not allow confident functional annotation (Table S3; sheets 1,2). In the absence of robust homology, these proteins remain classified as uncharacterized, as current annotation resources do not permit reliable assignment of biological roles. Heatmap analyses (Z-scores: –2 to 1 for total, –1 to 1 for nuclear) further confirmed the separation of young and mature tissues (Fig. 2C-D), with protein abundance generally higher in young tissues and declining in mature stipules and leaflets. Interestingly, uncharacterized proteins, distributed across distinct hierarchical clusters, displayed expression patterns that showed similarity and divergence from the overall trends observed in the combined proteome (Fig. 2E-F). Notably, a subset of total proteins (Fig. 2E, highlighted with a rectangle) accumulated more strongly in mature tendrils and stipules of Cooper than in their young counterparts or in Trapper. Similarly, nuclear protein clusters were preferentially enriched in Trapper’s mature tendril and petiole compared to Cooper (Fig. 2F, highlighted with a rectangle).

### Subset of proteins shared by different tissue types or genotypes

**Fig. 3:**
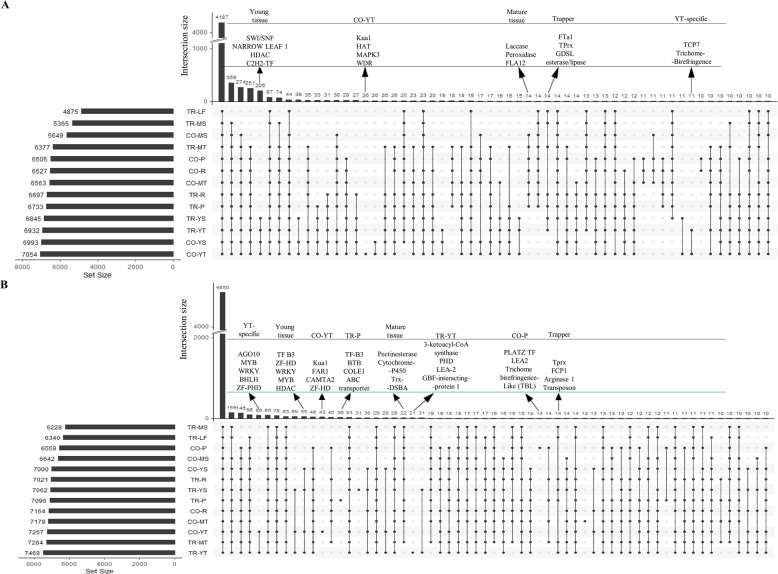
UpSet plots of proteins detected across pea leaf tissue types. **A** Total proteome and **B** nuclear proteome datasets are shown. Each row represents a leaf tissue type, with dots in columns indicating shared proteins across tissues. The minimum subset size was set to 10. Protein set sizes for each tissue type are shown on the left, while unique proteins specific to certain tissues or cultivars are displayed above the shared protein counts

Proteins specific to tissues or shared across tissues were identified using intersects from UpSet plots (Fig. 3A-B). Proteins detected in at least two of three replicates were considered present. The core proteome included 4,187 total proteins (Table S4, sheet 1) and 4,850 nuclear proteins (Table S5, sheet 1). In the total proteome, 206 proteins were specific to young tissues (S4, sheet 21), while only 14 were unique to mature tissues (S4, sheet 24). In contrast, 55 and 22 proteins were exclusive to young and mature tissues, respectively, in the nuclear proteome (S5, sheets 27, 24). Among the specifics in young tissue, C2H2 (Cys2/His2-type), HDAC (histone deacetylase), NARROW LEAF 1, and SWI/SNF (SWItch/Sucrose Non-Fermentable), were prominent in the total proteome, while HDAC, MYB (myeloblastosis), WRKY, ZF-HD (zinc finger-homeodomain) and TF-B3 (B3 domain containing transcription factor) were enriched in nuclear proteome. Focusing on tissue specificity independent of genotype, young tendrils accumulated 11 unique total proteins (S4, sheet 19) but as many as 86 nuclear proteins (S5, sheet 20). The important nuclear proteins common across cultivars were PHD (plant homeodomain), bHLH (basic helix-loop-helix), WRKY, MYB and AGO10 (argonaute 10), whereas TCP7 (teosinte branched 1/cycloidea/proliferating cell factor 7) and trichome-birefingence stood out in total proteome. Young tendrils also differed strongly by genotype, with Cooper possessing 26 total (S4, sheet 18) and 43 nuclear proteins (S5, sheet 19) undetected in Trapper, while Trapper showed 21 unique nuclear proteins (S5, sheet 21). Some of the regulatory nuclear proteins only detected in Cooper young tendrils were Kua1(KUODA1), FRS (FAR1-related sequence), CAMTA2 (calmodulin-binding transcription activator 2), and ZF-HD, whereas Trapper specifically accumulated PHD, LEA-2 (late embryogenesis abundant-2) and GBF-interacting protein 1 among others. In contrast to tendrils, stipules displayed fewer tissue- or genotype-specific signatures. Petioles, though morphologically similar between cultivars, contained 36 nuclear proteins unique to Trapper (S5, sheet 9) and 14 unique to Cooper (S5, sheet 7). Among the detectable proteins in Trapper nuclear proteome were ABC transporter, LAG1 longevity assurance 2, BTB domain containing protein and TF-B3, whereas Cooper had PLATZ-TF (plant AT-rich sequence and Zinc-binding protein), LEA2, and TBL (trichome birefringence-like). Rachis, defined as the main axis segment above leaflets or tendril branches (Fig. 1B), showed more common proteomic profiles between cultivars. Beyond tissue-specific patterns, clear genotype specificity emerged. Fourteen proteins each in total (S4, sheet 14) and nuclear (S5, sheet 15) proteomes were unique to Trapper, while 17 total proteins were unique to Cooper (S4, sheet 2), though none were detected in its nuclear proteome.

### Dynamics of protein changes across leaf tissue types

**Fig. 4:**
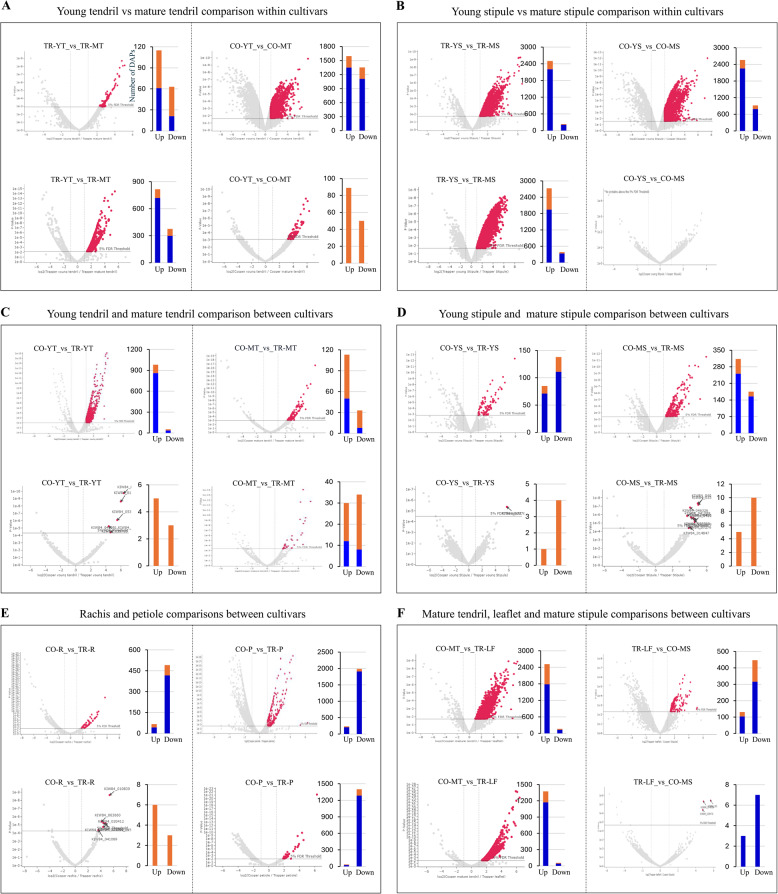
Differentially abundant proteins (DAPs) across leaf tissue-type comparisons. Volcano plots and bar charts summarize DAPs in total (top panels) and nuclear (bottom panels) proteomes for the indicated comparisons. The abbreviations denoting tissue-types and cultivars are defined in the plant material section of experimental procedures. The values with an FDR < 0.05 and a fold change ratio > 2 were considered as statistically significant. The thresholds are indicated by a horizontal line (5% FDR) and vertical lines (± two fold). Proteins up- and down-regulated are shown in red and gray (above the horizontal threshold line), respectively, while those with ≥ eight fold changes are highlighted in orange in bar graph. The y-axis in bar graph represents the number of DAPs

To explore dynamic changes in the total and nuclear proteomes, we performed differential abundance protein (DAP) analysis across leaf tissues of two pea cultivars. Trapper and Cooper leaves consist of seven and six distinct parts, respectively, yielding 78 possible pairwise comparisons for both proteomes (Table S6). Given the complexity and scale of the dataset, we focused on biologically informative contrasts: young versus mature tissues, homologous tissues across cultivars, and at times specific cases such as Trapper leaflets versus Cooper mature stipules, or Cooper mature tendrils versus Trapper leaflets. The latter is particularly interesting, as it reflects the proteomic consequences of the genetic mutation converting a leaflet into a tendril. Proteins were designated as differentially abundant if they showed at least a two-fold change at a 5% false discovery rate (Tables S7-S8). Tendril development revealed striking cultivar-specific patterns. In Cooper, ~ 3,000 total proteins and 140 nuclear proteins changed significantly between young and mature tendrils, with slightly more than half upregulated at the young stage (Fig. 4A right panels). By contrast, Trapper displayed only 178 total but 1,189 nuclear DAPs. Moreover, Trapper’s total DAPs often showed large fold changes (≥ eightfold), whereas Cooper’s nuclear DAPs exhibited stronger shifts (Fig. 4A left panels). Stipule development also involved extensive proteomic changes (3,000–3,500 total DAPs in both cultivars), but nuclear differences diverged: Cooper had none, while Trapper showed ~ 3,000 nuclear DAPs (Fig. 4B). Although abundant, stipule DAPs generally exhibited smaller fold changes compared to tendrils. Cross-cultivar comparisons highlighted additional contrasts. Young tendrils differed by 1,034 total DAPs between Cooper and Trapper, but only 146 at the mature stage (Fig. 4C). The opposite trend appeared in stipules, where mature tissues showed twice as many DAPs as young (488 vs. 223) (Fig. 4D). Nuclear DAPs were rare at early stages but often displayed ≥ eightfold differences, increasing substantially in mature tissue comparisons. In most homologous tissues (stipules or rachis), total DAPs outnumbered nuclear DAPs by 30–125 times. An exception was the petiole, where 2,200 total and 1,400 nuclear DAPs were detected (Fig. 4E).

The mutation replacing leaflets with tendrils was reflected in stark proteomic differences yielding about 2,700 total and 1,400 nuclear DAPs between Cooper mature tendrils and Trapper leaflets (Fig. 4F, left). In contrast, Trapper leaflets and Cooper stipules, which resemble somewhat morphologically, displayed far fewer differences (Fig. 4F, right). Remarkably, no significant nuclear DAPs were detected between Trapper leaflets and stipules, and only one total protein (serine/threonine kinase) was upregulated, underscoring that proteomic divergence is most pronounced between morphologically distinct tissues.

### Biological processes underlying proteomic changes in leaf tissue types

**Fig. 5:**
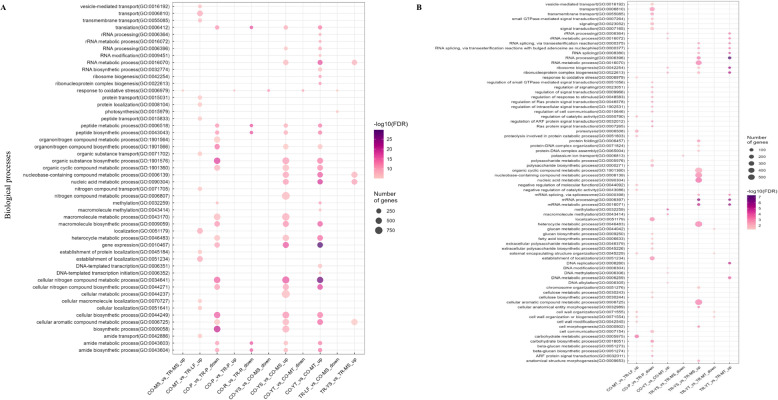
Enriched biological processes among differentially abundant proteins (DAPs) in total and nuclear proteomes. **A** The top 100 significantly enriched biological processes (sorted by *p*-value) among up- and down-regulated proteins in the total proteome for the indicated comparisons. The complete set of GO terms is shown in Fig. S2. **B** Significantly enriched biological processes in the nuclear proteome for selected comparisons. Dot color indicates the significance level (–log10 FDR-corrected *p*-value), while dot size represents the number of DAPs associated with each process. Comparisons with no significant enrichment are not shown

Leaf development is associated with changes in numerous biological processes [43]. To assess the changes in protein expression during leaf development, which are reflected in activation or suppression of diverse biological processes, GO enrichment analyses were performed on both total and nuclear proteomes. The analysis focused on the same key tissue-type comparisons described in the DAP analyses (Fig. 5A-B; Tables S9-S10). For simplicity, only the top 100 biological processes (*p* < 1.0e-05) from the total proteome are shown (Fig. 5A; Fig. S3), whereas all nuclear processes are displayed given their smaller number (Fig. 5B). Consistent with proteomic divergence, tendril development showed striking cultivar-specific patterns. In Cooper, young tendrils were enriched in processes related to nitrogen compound synthesis, gene expression, macromolecule biosynthesis and modification, and nuclear activity. Mature tendrils, by contrast, exhibited enrichment of carbohydrate metabolism, ATP synthesis, cell wall organization, oxidative stress response, and photosynthesis. In Trapper, most of these processes were absent or less pronounced. At the nuclear level, DNA and macromolecule methylation were prominent in Cooper young tendrils, whereas DNA and RNA metabolic processes dominated in Trapper. Notably, cell wall organization and biogenesis were downregulated in Trapper but not in Cooper.

The transition from young to mature stipules also engaged numerous processes in both cultivars (Fig. 5A). Shared enrichment included ATP and carbohydrate metabolism, amino acid biosynthesis, nucleotide metabolism, and cell wall organization. However, Cooper mature stipules uniquely displayed photosynthesis, stress, and oxidative stress responses. Cooper young tendrils were further characterized by upregulation of ncRNA processing, RNA splicing, peptide metabolism, and translation, whereas Trapper emphasized DNA replication and mRNA splicing/processing (Fig. S3). Although morphologically similar, petioles of the two cultivars exhibited dramatic divergence in enriched processes (Fig. 5A-B; Fig. 4E). In Trapper, processes related to macromolecule biosynthesis and metabolism (protein, lipid, polysaccharide), tRNA/rRNA/RNA/DNA modification, ribosome biogenesis, protein methylation, gene expression, translation, and ncRNA metabolism were strongly upregulated. Rachis tissues displayed limited differences, restricted mainly to peptide metabolism, ncRNA processing, and translation, which were downregulated in Cooper.

Comparisons of morphologically dissimilar tissues revealed especially large shifts in biological processes, paralleling extensive proteomic divergence. For instance, CO-MT versus TR-LF showed enrichment in protein transport, glycosylation, peptide and nitrogen compound metabolism, and carbohydrate biosynthesis in the total proteome, while nuclear enrichment included proteolysis, regulation of catalytic activity, cell wall modification, and carbohydrate metabolism. Similarly, CO-YT versus TR-LF was enriched in processes regulating gene expression, catalytic activity, protein transport/localization/modification, peptide transport, and lipopolysaccharide metabolism. Nuclear processes include RNA and mRNA processing, ribosome biogenesis, ncRNA-mediated gene silencing, nucleic acid metabolism, and DNA replication.

### Tissues– and cultivar -specific enrichment of leaf development proteins

**Fig. 6:**
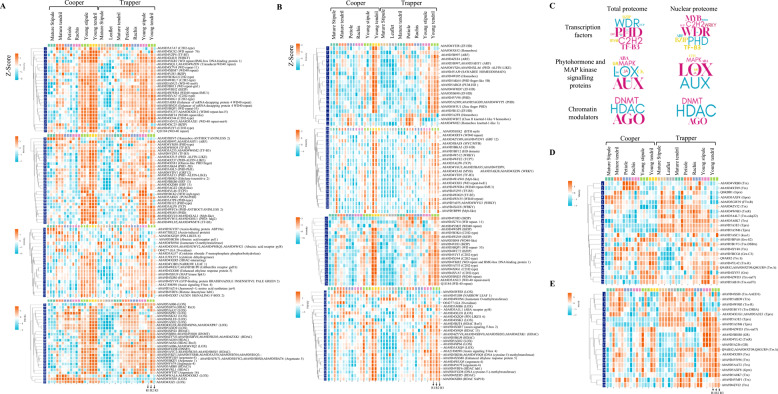
Differential abundance of orthologues of leaf development proteins in total and nuclear proteomes. **A-B** Heatmaps showing differential abundance of leaf development–related proteins in the total **A** and nuclear **B** proteomes. Because Mass Dynamics software provides protein names/IDs for up to 30 data points, multiple heatmaps were generated to accommodate the broader set, with rows numbered for reference. **C** Word cloud illustrating the frequency of leaf development proteins detected in the proteomic dataset. Larger and bolder names indicate a higher frequency of differentially abundant protein homologs. **D-E** Differential abundance patterns of selected Trx/Grx-related redox proteins in total **D** and nuclear **E** proteomes. For heatmaps, differentially abundant proteins (ANOVA, Benjamini–Hochberg corrected *p* < 0.05) were analyzed, and statistical comparisons incorporated MNAR-based imputation as described in Methods. Other details are the same as in Fig. 2

Leaf development, from initiation to senescence, is governed by a complex, multilayered regulatory network mediated by transcription factors (TFs), phytohormones, and epigenetic factors [44]. To explore the roles of orthologues of these regulators in pea leaves, their abundance patterns were analyzed, identifying TFs (twelve families), phytohormone-related proteins (seven families), epigenetic-related proteins (three families), lipoxygenase (LOX), and MAP-kinase family proteins (Table S11, sheets 1,2). Log₂ intensity-based heat map analysis revealed that both cultivars showed general enrichment of TFs and other proteins in young tendrils versus mature tendrils, though this was more pronounced in Cooper (Fig. 6A-B). The prominent TF families included PHD, WDR (WD-40 repeat), C2H2, AGO, TF-B3, ERF (ethylene response factor), MYB/MYC, WRKY, and bZIP (basic leucine zipper), with several showing stronger enrichment in Cooper young tendrils relative to Trapper. For example, C2H2 (rows 1, 26), WD repeat (rows 2, 5, 17, 18, 27), TF-B3 (rows 3, 32, 33, 55), WRKY (row 4), bZIP (row 25), PHD (rows 34, 35, 37, 40, 49, 50, 54), and MYB (row 44) displayed higher intensities in Cooper young tendrils, with many maintaining elevated levels into maturity (rows 1–2, 17–19, 25–26, 28, 30, 32–33, 44, 55). These enrichments were more prominent in the total proteome than in the nuclear fraction, with several changes reaching statistical significance (*p*adj < 0.05) (Table S11, sheet 5). In Cooper tendrils, enrichment of auxin, cytokinin, gibberellin (GA), and brassinosteroid signaling proteins was evident in the total proteome. Mature tendrils contrasted with Trapper and with Cooper’s younger tendrils in proteins such as auxin-binding (rows 56–58), abscisic acid receptor (rows 59, 61), and GA-related proteins (row 62). Young tendrils also showed elevated chromatin modulators, including AGO (rows 94–96, 99), HDAC (rows 85, 87–89, 97–98), and DNMT (DNA (cytosine-5) methyltransferase), with enrichment more pronounced in Cooper (Table S11, sheet 5, CO-YT vs CO-MT). Other notable differences involved NARROW LEAF 1 and the LOX family. Most LOX members, which are linked to development and stress signaling, in the total proteome (rows 78–84, 101–102) were enriched in young tendrils, particularly in Cooper, although one member belonging to a separate hierarchical cluster (row 76) followed the opposite trend. Some LOX (lipoxygenase) proteins (rows 79, 101, 102) remained abundant in Cooper mature tendrils. Additionally, tendril maturation was associated with significant upregulation of structural development proteins—laccase, expansin, TBL, and EXORDIUM-like 2, particularly in Cooper compared with Trapper (Table S11, sheet 5, CO-MT vs TR-MT).

As in tendrils, stipules generally exhibited higher TF abundance in the young stage than in the mature stage, especially in total proteome data. Exceptions included homeobox ANTHOCYANINLESS 2 (row 28) and auxin-binding protein, ABP19a (row 56). Proteins related to CK (cytokinin), AUX (auxin), GA (gibberellic acid), BR (brassinosteroid), ABA (abscisic acid), and JA (jasmonic acid) phytohormones were downregulated upon maturation (Fig. 6A-B), except for jasmonate O-methyltransferase (NP row 62), which was more abundant in mature stipules, particularly in Trapper. Cultivar differences included CO-MS enrichment of WDR (row 12), auxin-binding ABP19a (row 56), ANTHOCYANINLESS 2 (row 28), and LOX (row 101), whereas TR-YS accumulated more homeobox (NP row 17), HTH MYB-type (row 46), C2H2-type (TP row 10, and NP row 45), auxin signaling F-box 2 (TP row 75, and NP row 70), and LOX (NP row 64 and TP row 76). In total proteome analysis of petiole and rachis, Trapper generally showed higher TF abundance than Cooper, with prominent examples including WDR (row 5, 27), C2H2 (rows 10, 11), bZIP (row 9), oberon-like PHD (row 36), ERF 13 (row 42), abscisic acid receptor pyl8 (row 61), certain LOX variants (row 82, 90), and HDAC (rows 89, 92). Consistent with other tissues, LOX (rows 79, 101–102) and ANTHOCYANINLESS 2 (row 28) were upregulated in petiole of Cooper but not Trapper. While many TFs were more enriched in Trapper petiole and rachis than Cooper, leaflet tissue in Trapper, without a direct Cooper counterpart, generally showed reduced TF abundance, with only a few exceptions. The contrasting abundance patterns of several clusters of uncharacterized proteins across tissues and cultivars suggest that yet-unknown proteins may play important roles in organ development and function (Fig. 2E-F). Further investigation of this proteomic resource will be required to establish their specific contributions to leaf development.

Notably, members of WDR, PHD, TF-B3, bZIP and C2H2 TF families were more enriched than other TF families in both proteome datasets (Fig. 6C). Likewise, AUX, GA, ABA, JA, and ETH (ethylene) dominated the phytohormone-related group, while HDAC, AGO, and DNMT were noticeable among chromatin modulators. Additionally, LOX family members were prominently implicated in both total and nuclear proteins.

### Differential enrichment of Trx/Grx system proteins

The Trx/TrxR (thioredoxin/thioredoxin reductase) and GSH/Grx (glutathione/glutaredoxin) systems are central regulators of plant development and stress adaptation through redox homeostasis [17, 45, 46]. To assess their contribution to pea leaf development, we analyzed their distribution across total and nuclear proteomes. A total of 54 Trx/Grx-associated proteins were differentially abundant in the total proteome and 20 in the nuclear proteome (Table S11, sheets 3,4). Heatmap clustering revealed strong cultivar- and tissue-specific accumulation patterns (Fig. 6D-E). In Trapper, Trx (TP row 9; NP row 17) and two homologs of Tprx (thioredoxin-dependent peroxiredoxin; TP rows 10–11; NP rows 6–7) were consistently enriched.Trx-mrl7 (row 21) was preferentially accumulated in young tendrils of both cultivars, whereas Gprx (glutaredoxin-dependent peroxiredoxin) (row 3), FTrxR (ferredoxin-thioredoxin reductase; row 5), and Trx (row 6), predominated in Cooper mature tendrils (Table S11, sheet 5). Cooper tendrils were further distinguished by higher levels of Trxh (rows 17), while several nuclear Trx (row 19) and Tprx (rows 6–7) accumulated more strongly in Trapper tendrils (Fig. 6E). The total Trx (row 6), and Tprx (row 11) along with nuclear Trx (row 15) were highly abundant in young tendril of Trapper than Cooper.

Across other tissues, Trx proteins (rows 9, 11, 22) were generally more abundant in Trapper stipules (young and mature) compared with Cooper. Trapper leaflet and mature stipule, being morphologically similar, also exhibited comparable Trx/Grx abundance profiles. Interestingly, petioles and to some extent rachis showed striking cultivar-specific differences, particularly at the total proteome level (rows 1–2, 6–11), reinforcing earlier observations of petiole’s defiance to similar tissue protein patterns.

### Protein- protein interaction networks

**Fig. 7:**
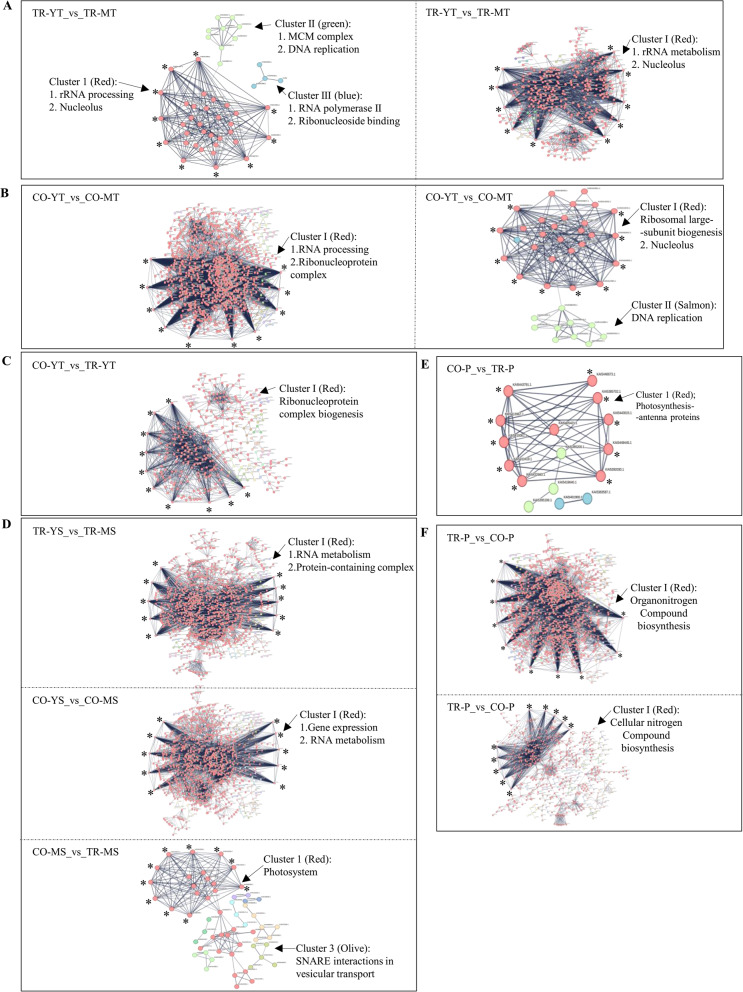
Protein–protein interaction network of up-regulated proteins across pea leaf tissue-type comparisons. **A-B** The protein-network identified in total (left panel), and nuclear (right panel) proteomes of indicated comparisons. **C**, **D** and **E** Protein network identified in total proteomes of selected comparisons **F** Predicted networks in total (top panel) and nuclear (bottom panel) proteome for Trapper vs. Cooper petioles. Key biological processes associated with specific clusters are shown with corresponding color codes in each network. The top 10 hub proteins in each network are indicated by stars. Details of cluster identities, color codes, and hub proteins are provided in Table S12 (total proteome) and Table S13 (nuclear proteome). Only networks with statistically significant interactions are displayed

To investigate the role of proteins in leaf tissue-type development, we constructed protein–protein interaction (PPI) networks of up-regulated proteins using STRING [47] from the total (Table S12) and nuclear (Table S13) proteomes of pea leaves (Fig. 7A-F). Young tendrils showed striking cultivar-specific contrasts: in Trapper, nuclear protein networks were denser than total proteins, while the reverse was true for Cooper (Fig. 7A, B). Networks in young tendrils of both cultivars were dominated by nuclear metabolism, including DNA replication, rRNA processing, and ribonucleoproteins, with Cooper further enriched in flavonoid and fatty acid biosynthesis (Tables S12 and S13, sheets 1–2). Ribosomal proteins namely BRIX (biogenesis of ribosomes) and BOP1 (block of Proliferation 1) emerged as central hubs in Cooper, whereas Trapper was characterized by WDR TFs, U3 snoRNAs, BRIX, and BMS1/TSR1 (ribosome biogenesis) proteins (sheets 3–4). Cross-cultivar comparison of young tendrils revealed further enrichment of WDR TFs, U3 snoRNAs, and ribosomal proteins as hub proteins in Cooper (Fig. 7C; Table S12, sheets 9–10).

In young stipules, dense PPI networks were detected in both cultivars, centered around RNA metabolism and protein-containing complexes, with WDR TFs, BRIX, and U3 snoRNAs as consistent hubs (Fig. 7D; Table S13, sheets 5-6). Mature stipules of Cooper showed enrichment in photosystem proteins, SNARE-mediated vesicle transport, and phenylpropanoid biosynthesis, with chlorophyll a–b binding proteins and photosystem I as major hubs (Table S12, sheets 15-16). In petioles, where most total proteins were downregulated in Cooper compared to Trapper, networks were less dense (Fig. 7E; Table S12, sheet 11). Nonetheless, Cooper petiole networks highlighted photosynthesis antenna proteins with chlorophyll a-b binding protein and photosystem I as hub proteins (Table S12, sheet 12), as in stipules. Trapper petiole, on the other hand, was enriched in secondary metabolism, ER–Golgi transport, and redox-related proteins (Fig. 7F; Table S12, sheets 19-20). Across datasets, ribosomal proteins consistently emerged as prominent hub nodes (TP sheet 20; NP sheet 10).

## Discussion

A deeper understanding of leaf developmental biology not only holds promise for improving crop performance but also provides critical insights into broader plant developmental processes, laying a foundation for advancements in agriculture and forestry. Despite significant progress in leaf developmental biology [3], much remains to be understood about the specific roles and interactions of individual proteins, particularly in compound leaves. This study on two contrasting leafed and semi-leafless pea cultivars, offers a comprehensive overview of the total and nuclear proteomes across different leaf parts, identifying approximately 7,800 total proteins and 8,600 nuclear proteins and analyzing their abundance patterns in various leaf tissue types. Comparative analysis revealed that approximately 54% of total proteins constituted the core proteome, while 57% of nuclear proteins were shared across all tissue types (Fig. 3A-B). Despite the larger number of nuclear proteins, the DAPs in all tissues showed fewer changes in the nuclear proteome (36.7%) than in the total proteome (78.2%), suggesting a conserved response in nuclear protein regulation. Principal component analysis of both proteomes distinguished young from mature tissues regardless of cultivar. Notably, total proteins common to young tendrils and stipules were ~ 15-fold more abundant than in mature tissues, while nuclear proteins were twice as enriched (Fig. 3), underscoring the regulatory role of nuclear and other proteins during tissue development. These findings are consistent with the role of spatiotemporal protein accumulation in leaf developmental processes [44]. The thousands of proteomic changes distinguishing Cooper’s leaf phenotype highlight intensive proteome reprogramming, in line with reports that single-gene mutations can alter hundreds of proteins [48, 49]. Although cultivar-specific differences were evident, the results point to a strong influence of the *af* locus on tendril proteomes.

**Fig. 8:**
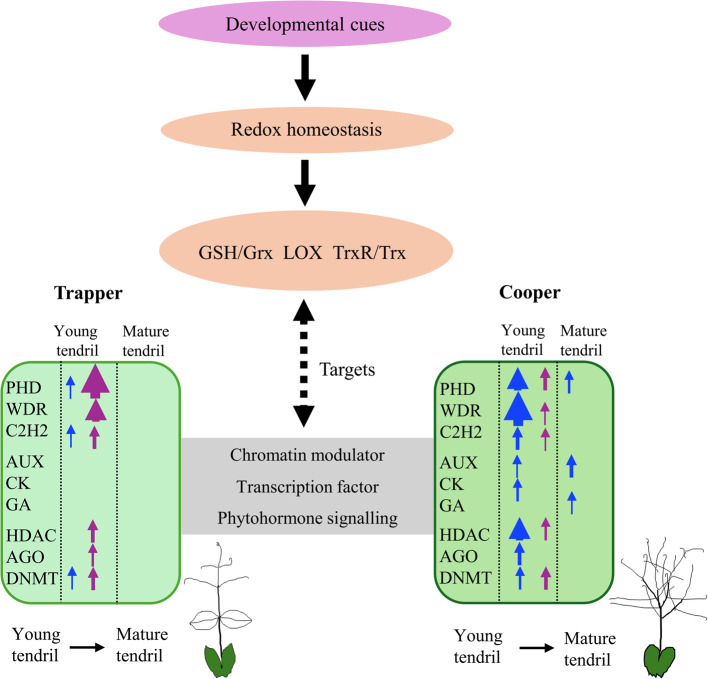
Hypothetical model illustrating the roles of cellular redox status, transcription factors, phytohormones, and chromatin modulators in tendril development of the Cooper and Trapper cultivars. Solid black arrows indicate events supported by the literature, whereas dotted double-headed arrows denote putative interactions. Green-panel arrows represent higher abundance of the indicated proteins in comparisons between young and mature tendrils. Blue arrows indicate protein abundance in the total proteome, and purple arrows indicate abundance in the nuclear proteome. Arrow thickness reflects the frequency of higher abundance of homologous proteins in the associated tissues. The supportive protein fold changes and significance levels are provided in Supplementary Table S15

Trapper develops residual tendrils, whereas Cooper forms highly branched tendrils (Fig. 1A-B). In Cooper, thousands of DAPs distinguished young and mature tendrils, while Trapper exhibited only 5–6% as many. Nuclear contributions were disproportionately higher in Trapper (nuclear/total DAPs = 6.7) than in Cooper (0.05), underscoring the regulatory emphasis of nuclear proteins in tendril development. Several transcription factors including TCP7, AGO10, MYB, WRKY, bHLH, and PHD-type were detected exclusively in young tendrils, regardless of cultivar, indicating their conserved role in early tendril development. However, the significant abundance of WDR, bZIP, WRKY, C2H2, Nal1 (NARROW LEAF 1) and PHD in Cooper, along with reduction in MAPK (mitogen-activated protein kinase) (Table S11, sheet 5, CO-YT vs TR-YT), suggest a key contribution to divergent developmental trajectories, as many of these proteins are known regulators of leaf development. For example, TCP7 and ORANGE control leaf size [50], AGO10 establishes leaf polarity [51], bZIP regulates leaf epidermal cells [52], Nal1 regulates leaf vein patterning or shape [53], C2H2 homologs convert pea leaflets into tendrils [12], and homeobox TFs regulate compound leaf morphogenesis [54]. Higher abundance of PHD, WDR, C2H2, TF-B3, and bZIP proteins in maturing tendrils of Cooper indicates a continuum of development from the young stage. Although B3 TFs are generally linked to seed development and embryogenesis, their consistent enrichment in tendrils across both proteomes suggests a novel role in leaf development. Hormonal signaling also contributed to tendril divergence. Cytokinin, GA, brassinosteroid, LOX variants, ARF (auxin response factor), auxin-induced protein, and auxin binding proteins were enriched in Cooper, while jasmonate O-methyltransferase was upregulated in Trapper. Although JA is generally viewed in defense signaling, its altered metabolism also affects leaf development in Arabidopsis [55]. Auxin is a major regulator of simple and compound leaf morphogenesis [56], with GA acting downstream of auxin during leaf growth [57]. Higher amount of GA 20-oxidase and several LOXs in Cooper mature tendril, and jasmonate O-methyltransferase in Trapper in both proteomes signify their antagonistic roles in controlling cell division, expansion and elongation of mature form of tendrils [58]. Moreover, Cooper-specific accumulation (Fig. 3B) of CAMTA2, a calmodulin-binding transcriptional activator involved in stress and developmental regulation [59], and FAR1, a regulator of branching in Arabidopsis [60], suggest additional layers of regulatory complexity in tendril branching and morphology. Chromatin remodelling also appeared to have facilitated the enhanced transcriptional activity in Cooper, as indicated by the upregulation of HDAC, AGO4, and DNMT in young tendrils. Along with regulatory proteins, phytohormones, and chromatin methylation/acetylation, the cellular redox milieu also contributed to the biological events of different tendril phenotypes as indicated by distinct enrichment of redox proteins in the two cultivars. For instance, Kua1, which is known to regulate leaf cell expansion under redox control [20] was detected only in Cooper young tendrils (Fig. 3A-B). Trapper-specific enrichment of Tprx and Trx proteins in both proteomes employ their roles in detoxifying H_2_O_2_ and redox-signalling mediated control of other proteins function [17]. Furthermore, their higher abundance in Trapper’s mature stipules and leaflets than other tissue-types indicates that these processes are more prominent in these tissues. However, enrichment of total Grx-S2/C3 and Trx proteins in Cooper than Trapper suggests differential mechanism of redox homeostasis between both the cultivars. Beyond developmental regulation, enrichment of pathways related to gene expression, biosynthesis, carbohydrate metabolism, ATP synthesis, photosynthesis, and cell wall organization (Fig. 5A-B) supported tendril structure and function in Cooper. Notably, the significantly higher presence in mature tendrils of laccase, expansin, fasciclin-like arabinogalactan 12, pectinesterase, and peroxidase enzymes (Table S11, sheet 5, CO-YT vs CO-MT) that are involved in lignin biosynthesis, cell wall loosening, and enrichment of cytoskeleton, and tubulin complex assembly ensured the structural requirements of tendrils for their grasping role. Regulatory proteins such as EXORDIUM-like 2 (cell expansion; [61] and TBL (cell wall polysaccharide acetylation; [62]) also supported structural reinforcement in maturing tendrils. A significant up-regulation of certain transcription factor, phytohormonal signaling, chromatin modulator and redox pathway proteins in Cooper mature tendril than Trapper leaflet in both the proteome studies contributes to the divergent growth and development of two tissues (Table S14; sheets 1,2). Based on our findings, we propose a hypothetical regulatory framework that may underlie the observed differences in pea leaf morphology (Fig. 8; Table S15; sheets 1–4). This model highlights a potential role for cellular redox balance, predominantly mediated through thioredoxin (Trx), glutaredoxin (Grx), and lipoxygenase (LOX) pathways, in modulating leaf developmental programs. We hypothesize that developmental signals may be integrated through redox homeostasis, thereby influencing the activation or repression of key regulatory proteins, potentially via alterations in chromatin-modifying complexes. Importantly, this model is derived from associative proteomic evidence and should therefore be interpreted with caution. Experimental validation will be required to substantiate these proposed regulatory interactions and to establish causal relationships. Also, it is important to note that we attempted to capture proteomic dynamics at the earliest possible stages of tissue differentiation, an inherent gap remains between the shoot apical meristem (SAM) primordia stage and the differentiated tissues analyzed here. Consequently, distinct regulatory proteins or alternative accumulation patterns at earlier stages cannot be ruled out. Future studies employing single-cell or ultra-low-input proteomic approaches at earlier developmental time points will be critical for resolving cellular heterogeneity and developmental trajectories as controlled by the regulatory entities during compound leaf formation.

The transition from young to mature stipules involving a large number of DAPs, with a higher proportion of them belonging to total proteome, was more aligned with the Cooper tendril development rather than Trapper. Higher abundance of regulatory proteins in young stipules paralleled tendril development, yet differences between cultivars were less pronounced in stipules than in tendrils, as indicated by fewer number of DAPs (Fig. 4D). Enrichment of WDR, C2H2, bZIP, Oberon-like PHD, ARF, ERF13, LOX variants, redox proteins, TCP2, and HDAC at the young stage emphasized the importance of these proteins in leaf development. Stipule-specific accumulation of TORNADO 2, which regulates early leaf patterning [63] was also observed. Despite phenotypic similarity, cultivar differences emerged: Cooper’s mature stipules showed higher abundance of wall-associated kinases (cell expansion regulators) [64] and SWEETIE proteins (sugar flux for wall growth) [65]. Likewise, in tendrils, dense PPI networks were detected in stipules across cultivars, centered on RNA metabolism and protein complexes. In mature stipules, Cooper showed enrichment of photosystem hubs, including chlorophyll a–b binding proteins and photosystem I (Fig. 7D). Given that stipules are Cooper’s only flat surface tissue, compared to Trapper’s additional leaflets, this may underscore their greater photosynthetic contribution in Cooper. The presence of a cluster with photosynthesis-antenna proteins in Cooper petiole (Fig. 7E) further highlights the compensation of photosynthetic capacity through non-leafy surfaces.

Comparisons across tissues revealed a trend where high morphological similarity corresponded to fewer DAPs (Fig. 4D), and greater phenotypic divergence correlated with more DAPs (Fig. 4F, left panels). Following this trend, there were only a few hundred DAPs with only less than ten detected at the nuclear level in the rachis comparison. In sharp contrast, however, petiole comparison revealed a very large number of DAPs in both the proteomes (Fig. 4E- Right panels). The large number of nuclear proteins (> 1,300) upregulated in Trapper petiole points to an extensive regulatory role. Such a scale of nuclear proteome influence was otherwise only observed in Trapper tendrils (Fig. 4A, left panels). Enrichment of WDR, bZIP, certain LOX proteins, PHD, ERF13, and reduced homeobox ANTHOCYANINLESS, together with a wide range of biological processes including RNA metabolism and upregulated chlorophyll biosynthesis, paralleled the patterns seen in young vs. mature tissues. This unusual behavior of the Trapper petiole was initially puzzling, but a closer examination of sampling time and developmental stage offered important clues. The sampling was done when the Trapper had one pair of leaflets, but two more pairs formed subsequently (Fig. 1C). The petiole seems to have served as a regulatory protein reservoir enabling additional leaflet development. A similar enrichment pattern in the rachis reinforces this interpretation. Interestingly, the accumulation of regulatory proteins was evident in the total proteome but not in the nuclear proteome, strengthening the notion of a reservoir function. This aligns with a recent report showing that certain transcription factors remain sequestered in the cytoplasm in inactive states and only translocate to the nucleus under specific environmental stimuli [66]. Although further evidence is needed to confirm role-specificity, these findings highlight the importance of WDR, C2H2, bZIP, PHD, TF-B3, and redox-regulatory proteins (Trx/Grx), together with chromatin acetylation/methylation dynamics and phytohormone signaling, in regulating compound leaf development. We identified several candidate regulatory proteins, including transcription factors, redox homeostasis components, and phytohormone-related proteins, that exhibit tissue-specific and differentially abundant patterns across pea leaf tissue-types. Functional validation of these candidates will be essential to establish their precise roles in compound leaf and tendril development. Such validation could be achieved through targeted overexpression, knockdown (RNAi), or knockout approaches using CRISPR-based genome editing, providing mechanistic insight beyond the associative proteomic evidence presented here. Leaf development proceeds through initiation, differentiation, and expansion phases. Leaf primordia formed at the SAM represent the earliest stage of this process and involve multilayered regulation mediated by phytohormones and diverse regulatory proteins [3]. A large number of DAPs were predicted to be networked, with nuclear metabolism central in both cultivars. Nevertheless, distinct hub proteins in PPI could be among the important drivers of two phenotypes. Given that hundreds of proteins in both total and nuclear proteomes remain uncharacterized, their distinct abundance patterns across tissue types and cultivars further suggest potential roles in organ development and function. Closer investigation of these proteins will be essential, as they may uncover previously unknown regulators of leaf development.

In summary, this study reveals how nuclear and total proteomes differentially contribute to tissue-specific development in two contrasting cultivars. The markedly distinct patterns of protein accumulation in tendrils emphasize the central role of regulatory proteins, hormones, redox signaling, chromatin remodeling, and cellular metabolism in shaping organ identity and function. The *af* locus mutations was the major determinant of tendril proteomes, with Cooper exhibiting extensive proteome reprogramming consistent with its highly branched phenotype, while Trapper displayed a more conserved nuclear regulatory emphasis. Several regulatory proteins appear to be key drivers of leaf development, and the protein–protein interaction networks with unique hub proteins underscore the importance of coordinated biological events in shaping organ function. The high-throughput proteomic data presented here would be a valuable resource to unravel the significance of complex interactions with emerging AI (artificial intelligence) tools to advance our understanding of leaf development.

## Conclusions

Leaf architecture is central to canopy structure, photosynthesis-driven productivity, specialized roles and stress adaptation in crops. This study provides an exceptional and valuable total and nuclear proteome information in that accumulated in different tissue-types of pea leaves of leafed (Trapper) and semi-leafless type (Cooper) pea cultivars. We detected 7,761 total proteins, and 8,580 nuclear proteins resulting identification of 6,068 total and, 3,149 nuclear differentially abundant proteins. Intersect plot analysis identified 4,187 total proteins and 4,850 nuclear proteins as core proteomes along with several protein contrasts detected in particular tissue-types. The comparison across Cooper and Trapper leaf tissue-types revealed major proteome changes during development of young and mature tissues, leaflet, and petiole. The biological processes related to transcriptional and post-transcriptional control were enriched in young tendrils, while localization, transport, protein modification, and cell wall related GO terms were enriched in mature tendrils of Cooper. Additionally, young stage of tendril and stipule development showed up-regulation of transcription factors, hormone responsive, redox, and chromatin modulator proteins than other leaf tissue-types. Further, PPI analysis revealed tissue-specific protein-network governing their development. The young tendril protein-network was enriched with clusters of proteins involved in RNA processing and DNA replication than their mature form of development. By mapping nuclear and total proteomes across compound leaf tissues in pea, this work reveals environment of regulatory proteins, biological processes, and protein–protein interactions that shape organ development and function, thus providing a foundation for systems-level approaches to advance molecular understanding and future crop improvement.

## Supplementary Information

Supplementary Material 1: Fig. S1 Nuclei quality analysis and PCR analysis of *PsPALM1a/b* gene. Representative images of nuclei isolated from young tendril of Cooper A, and petiole of Trapper B. The nuclei were stained with DAPI and visualized on eclipse TE300 inverted microscope. C Full-length original agarose gel image showing PCR amplified *PsPALM1a/1b *gene fragments from genomic DNA of Cooper and Trapper pea cultivars. The red colored rectangular shape denotes the cropped image shown in Fig. 1D. Lane 1 to 20 represents PCR amplification of other candidate genes and is not related to current study. The *PsTubulin *PCR was performed on genomic DNA of Cooper and Trapper pea cultivars to check their DNA quality. The 100 base pair marker was loaded for size verification and non-template control (NTC) was used as negative control. Fig. S2 Sample intensity correlation plot and protein count. A Protein intensity correlation matrix of total proteome and, B nuclear proteome. The scale bar red to blue color represents low to higher sample correlation values. C Bar chart of protein count in replicates of each tissue-types from total proteome, and D nuclear proteome. The y-axis represents protein count and x-axis specify sample (tissue and genotype) details. The bar color represents specific tissue-types used in this study. Fig. S3 Significant biological processes in up and down-regulated proteins identified in different tissue-type comparisons from total proteome analysis. The dot color denotes the significant enrichment, and the dot size specifies the number of DAPs related to the biological process. Details of these biological processes are provided in Table S9. The contrasts without significant GO-terms were not included in the Figure. Table S1 All identified precursor peptides and protein groups detected in total proteome study. Table S2 All identified precursor peptides and protein groups detected in nuclear proteome study. Table S3 List of differentially abundant protein identified through ANOVA in total and nuclear proteome data. Table S4 List of total proteins identified as genotype-specific, tissue-specific regardless genotype, and tissue-specific within each genotype. Table S5 List of nuclear proteins identified as genotype-specific, tissue-specific regardless genotype, and tissue-specific within each genotype. Table S6 Details of 78 pairwise comparison between Cooper and Trapper tissues and number of differentially abundant proteins identified in total and nuclear proteomes. Table S7 List of DAPs identified in the total proteome comparisons between Cooper and Trapper tissue-types. Table S8 List of DAPs observed in the nuclear proteome comparisons between Cooper and leaf Trapper tissue-types. Table S9 The enrichment of GO terms in DAPs from total proteome study. Table S10 The enrichment of GO terms in DAPs from nuclear proteome study. Table S11 Orthologues of leaf development, and Trx/Grx system proteins identified in total and nuclear proteomes. Table S12 Details of protein-network clusters and hub proteins identified in up-regulated proteins of total proteome data. Table S13 Details of protein-network clusters and hub proteins identified in up-regulated proteins of nuclear proteome data. Table S14 List of key differentially abundant proteins identified in total and nuclear proteomes of Cooper mature tendril and Trapper leaflet comparisons. Table S15 List of potential candidate proteins used to generate regulatory model governing pea young and mature tendrils development.

## Data Availability

All data used in this study are provided in the supporting information of this article. The mass spectrometry data have been submitted to the ProteomeXchange Consortium under dataset number PXD073277.
