# Supplementary material for: Integrative nuclear and total proteomics of compound leaves in semi-leafless and leafed pea cultivars reveal regulatory cues in leaf development
Source: BMC Plant Biol. 2026 Mar 4;26:824. doi: 10.1186/s12870-026-08381-5 (PMC13154526; doi:10.1186/s12870-026-08381-5)
Supplement: Supplementary file 1 — Supplementary Material 1: Fig. S1 Nuclei quality analysis and PCR analysis of PsPALM1a/b gene. Representative images of nuclei isolated from young tendril of Cooper A, and petiole of Trapper B. The nuclei were stained with DAPI and visualized on eclipse TE300 inverted microscope. C Full-length original agarose gel image showing PCR amplified PsPALM1a/1b gene fragments from genomic DNA of Cooper and Trapper pea cultivars. The red colored rectangular shape denotes the cropped image shown in Fig. 1D. Lane 1 to 20 represents PCR amplification of other candidate genes and is not related to current study. The PsTubulin PCR was performed on genomic DNA of Cooper and Trapper pea cultivars to check their DNA quality. The 100 base pair marker was loaded for size verification and non-template control (NTC) was used as negative control. Fig. S2 Sample intensity correlation plot and protein count. A Protein intensity correlation matrix of total proteome and, B nuclear proteome. The scale bar red to blue color represents low to higher sample correlation values. C Bar chart of protein count in replicates of each tissue-types from total proteome, and D nuclear proteome. The y-axis represents protein count and x-axis specify sample (tissue and genotype) details. The bar color represents specific tissue-types used in this study. Fig. S3 Significant biological processes in up and down-regulated proteins identified in different tissue-type comparisons from total proteome analysis. The dot color denotes the significant enrichment, and the dot size specifies the number of DAPs related to the biological process. Details of these biological processes are provided in Table S9. The contrasts without significant GO-terms were not included in the Figure. Table S1 All identified precursor peptides and protein groups detected in total proteome study. Table S2 All identified precursor peptides and protein groups detected in nuclear proteome study. Table S3 List of differentially abundant protein [file 12870_2026_8381_MOESM1_ESM.zip › Fig. S1.pdf]

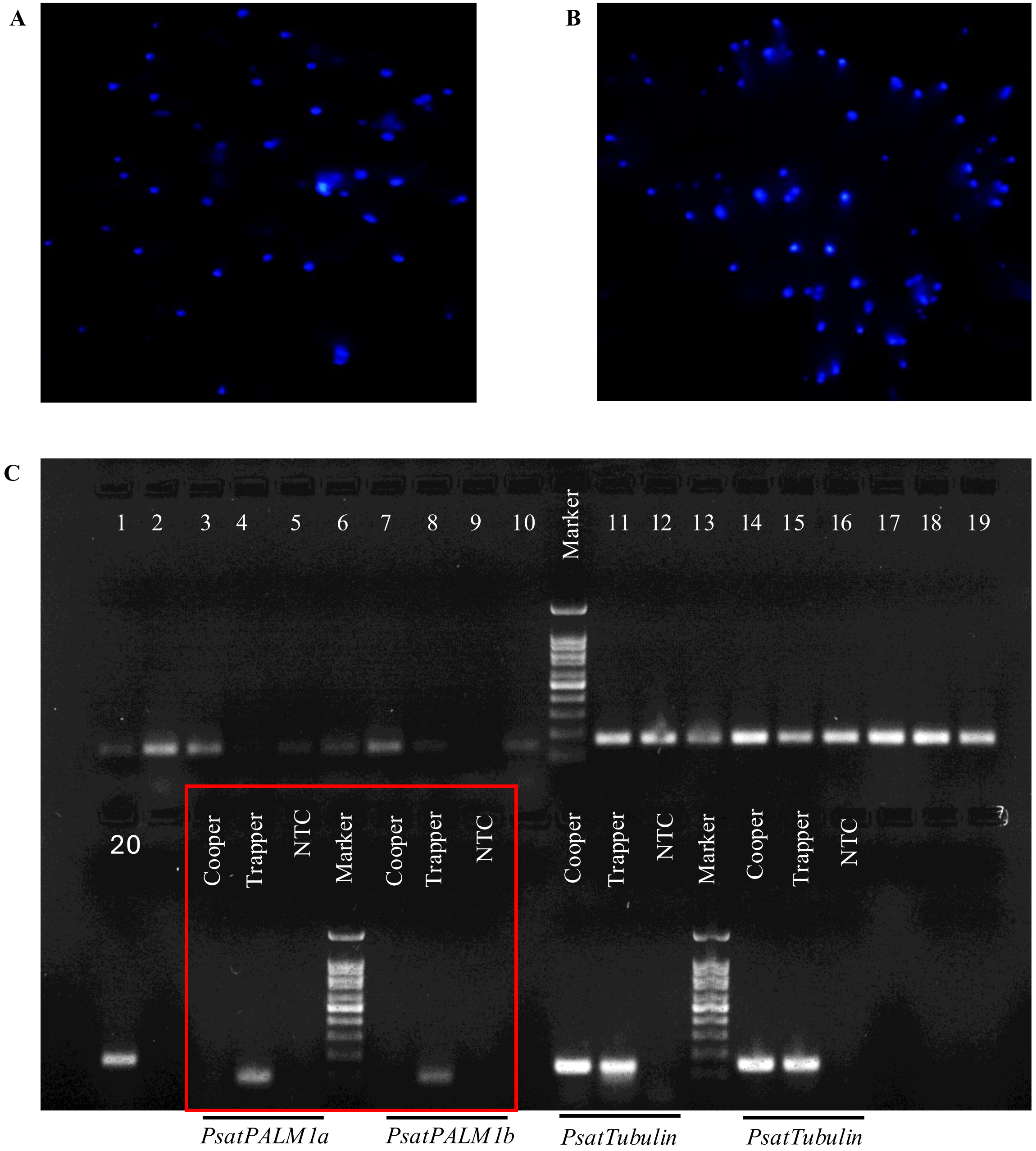

Fig. S1. Nuclei quality analysis and PCR analysis of *PsatPALM1a/b* gene. Representative images of nuclei isolated from young tendril of Cooper **A**, and petiole of Trapper **B**. The nuclei were stained with DAPI and visualized on eclipse TE300 inverted microscope. **C** Full-length original agarose gel image showing PCR amplified *PsatPALM1a/1b* gene fragments from genomic DNA of Cooper and Trapper pea cultivars. The red colored rectangular shape denotes the cropped image shown in Fig. 1D. Lane 1 to 20 represents PCR amplification of other candidate genes and is not related to current study. The *PsatTubulin* PCR was performed on genomic DNA of Cooper and Trapper pea cultivars to check their DNA quality. The 100 base pair marker was loaded for size verification and non-template control (NTC) was used as negative control.
